# Determination of 25 Organophosphate Ester Flame Retardants in Soils by Accelerated Solvent Extraction–Ultra-High Performance Liquid Chromatography

**DOI:** 10.3390/toxics14090763

**Published:** 2026-08-26

**Authors:** Ban Cao, Yinjun Shi, Zuguo Hu, Mingli Ye

**Affiliations:** 1 Zhejiang Institute of Geosciences, Hangzhou 310007, China; caob1981@163.com; 2 Qiantang Institute of Geology, Hangzhou 310007, China; 3 Zhejiang Diwu Inspection Group, Hangzhou 310007, China; 4 Zhejiang Academy of Special Equipment Science, Hangzhou 310000, China; 5 Key Laboratory of Special Metal Structure Material Testing and Evaluation, State Administration for Market Regulation, Hangzhou 310000, China; 6 Zhejiang Key Laboratory of Special Equipment Safety Technology, Hangzhou 310000, China; 7 College of Biological and Environmental Engineering, Zhejiang Shuren University, Hangzhou 310015, China

**Keywords:** organophosphate esters (OPEs), liquid chromatography-tandem mass spectrometry, accelerated solvent extraction, soil

## Abstract

Organophosphate esters (OPEs) are commonly used flame retardants and plasticizers, which can easily be released into the soil environment and have potential hazards such as neurotoxicity and developmental toxicity. Establishing an efficient and sensitive detection method plays a crucial role in soil pollution assessment. Currently, there have been numerous studies on the detection of OPEs in soil using ultrasonic extraction and solid-phase extraction, while relatively few studies have focused on the analysis of multiple OPEs in soil by accelerated solvent extraction combined with d-SPE clean-up and ultra-high performance liquid chromatography–tandem mass spectrometry. The d-SPE purification method eliminates the need for column passage, shortens sample preparation time, and avoids the risk of background contamination from OPEs that may be introduced by SPE. This study developed a liquid chromatography–tandem quadrupole mass spectrometry (LC-MS/MS) method for the determination of 25 OPEs in soil, and systematically optimized the pretreatment and instrumental analysis conditions. Accelerated solvent extraction was used for pretreatment, and dichloromethane–methanol (1:1, *V*/*V*) was determined as the optimal extraction solvent. A mixed adsorbent of N-propyl ethylenediamine (PSA) and C18 was selected for dispersive purification, effectively removing matrix interference and improving recovery rates. The mass spectrometry parameters such as collision energy and declustering voltage were optimized, significantly enhancing ion response intensity and detection sensitivity. The method showed good linearity within the concentration range of 1–100 ng/mL, with correlation coefficients all greater than 0.994. The spiked recovery rates ranged from 70.6% to 111% with a relative standard deviation (RSD) of 1.2–11.8%. The precision and accuracy met the requirements for environmental sample analysis. The method was applied to the detection of actual soil samples, and the results were stable and reliable. This method is simple to operate, highly sensitive, and widely applicable, providing reliable technical support for the pollution monitoring, source tracing, and ecological risk assessment of OPEs in soil.

## 1. Introduction

Organophosphate esters (OPEs) are synthetic derivatives of phosphoric acid, featuring a central phosphate group in which the three hydrogen atoms are replaced by various organic substituents. Depending on the chemical nature of these substituents, OPEs are commonly classified into three major categories: alkyl OPEs (e.g., tributyl phosphate, TnBP), halogenated OPEs (e.g., tris(2-chloroethyl) phosphate, TCEP; tris(2-chloropropyl) phosphate, TCPP), and aryl OPEs (e.g., triphenyl phosphate, TPhP).

Owing to their high flame-retardant efficiency, good plasticity, and low production cost, OPEs are extensively employed as plasticizers and flame retardants in packaging materials, furniture, electrical and electronic equipment, and building materials [1,2,3]. In most applications, OPEs are incorporated as additive-type flame retardants rather than chemically bonded to the material matrix, which facilitates their release into the environment via abrasion, volatilization, leaching, and other pathways throughout the product lifecycle—from manufacturing and use to disposal. Once released into the atmosphere, organophosphate esters (OPEs) are distributed in three main forms: gaseous, aerosol-associated, and adsorbed onto particulate matter. These atmospheric OPEs are subsequently transferred to water bodies and soils via dry and wet deposition processes. Meanwhile, OPEs present in soils are not permanently retained; they can be mobilised by rainwater runoff and volatilise back into the atmosphere, leading to secondary contamination of both water and air environments. This inter-compartmental exchange highlights the dynamic cycling of OPEs across environmental media.

As industrial innovation progresses, novel OPEs with diverse structural properties continue to be synthesized and put into large-scale use. Consequently, as emerging contaminants, organophosphate ester flame retardants have drawn increasing research interest concerning their environmental occurrence and fate. To date, OPEs have been ubiquitously detected in various environmental compartments, including water [4,5,6,7], soil [8,9], sediments [10,11], dust, and air [12,13], and have even been found in human biological matrices such as urine [14,15], blood [16,17], breast milk [18,19], and placenta [20]. Furthermore, their neurotoxicity, reproductive toxicity, and bioaccumulation potential [21,22] not only threaten ecosystem health but also pose potential risks to humans through food chain transfer. Human exposure can be further exacerbated via the inadvertent ingestion of soil (particularly in children through hand-to-mouth behaviour), inhalation of resuspended soil particles, or uptake by crops, which subsequently introduce OPEs into the food web and thereby amplify exposure risks.

Certain areas are particularly vulnerable to OPE contamination, including landfills, electronic waste recycling sites and industrial parks (especially those involved in plastic, textile, and foam manufacturing). The dominant sources of OPEs in soils can be categorised into four primary pathways. First, atmospheric deposition—transports particle-bound OPEs originating from combustion, vehicular traffic, and industrial emissions. Second, direct application of OPE-containing consumer and industrial products, such as flame-retarded textiles, upholstery, building materials, and agricultural plastic films (e.g., greenhouse films), releases these compounds into surrounding soils through abrasion, weathering, and disposal. Third, landfill leachate and surface runoff from waste disposal sites serve as significant point sources. Collectively, these pathways determine the spatial distribution and contamination levels of OPEs in diverse soil environments. Fourth, agricultural practices, including the application of sewage sludge/biosolids and irrigation with reclaimed wastewater, also contribute to OPE accumulation in farmland soils.

Compared with water samples, the analysis of soil matrices poses greater technical challenges. Soil is inherently complex, containing substantial organic matter, humic acids, and clay minerals, which strongly adsorb target analytes onto particle surfaces or trap them within aggregates, rendering extraction particularly difficult. Moreover, abundant co-extracted interferents can severely compromise subsequent instrumental analysis, leading to pronounced matrix effects and reduced accuracy.

Commonly employed techniques for extracting OPEs from solid samples include Soxhlet extraction, ultrasonic extraction, microwave extraction, and accelerated solvent extraction (ASE). Several studies have explored various methods for OPE determination in soil: Liu et al. [23] used ultrasonic extraction; Liu et al. [24] employed vortex-assisted ultrasonic extraction to recover 8–9 common OPEs from sediments; Luo et al. [25] applied pressurized solvent extraction to 13 OPEs in soil; and García-López et al. [26] used the same technique for 7 OPEs in sediments.

Collectively, these studies indicate that existing methods cover only a limited number of OPE congeners, falling short of the growing demand for simultaneous multi-analyte detection in environmental monitoring. Therefore, there is an urgent need to develop reliable analytical protocols that enable rapid, sensitive, and selective identification of these emerging contaminants in complex sample matrices.

Compared with conventional techniques such as Soxhlet, ultrasonic, and microwave extraction, accelerated solvent extraction (ASE) offers substantial advantages. The method automates the extraction of target compounds from solid or semi-solid samples using organic solvents under elevated temperature and pressure. At high temperature, analyte solubility increases, intermolecular interactions (e.g., van der Waals forces and hydrogen bonds) between solutes and the matrix are weakened, desorption kinetics accelerate, and solvent viscosity decreases while diffusion coefficients rise—collectively promoting solvent penetration and extraction efficiency. Meanwhile, high pressure raises the solvent boiling point, maintaining it in a liquid state at elevated temperatures and enabling rapid solvent delivery. Although extraction is conducted at high temperature, the exposure time is kept below 10 min, effectively preventing thermal degradation and ensuring satisfactory recoveries even for volatile and labile compounds. With its high efficiency, energy savings, and full automation, ASE has been widely adopted in environmental monitoring, food safety, and pharmaceutical analysis.

The instrumental analysis of OPEs is primarily governed by their physicochemical properties. Many OPEs are sufficiently volatile, making gas chromatography (GC) and gas chromatography–mass spectrometry (GC–MS) conventional detection techniques [27,28,29,30]. However, certain OPEs with higher molecular weights or thermal instability pose challenges for GC–MS, such as thermal decomposition or poor vaporization. In such cases, liquid chromatography–mass spectrometry (LC–MS) offers a more suitable alternative, particularly liquid chromatography–tandem mass spectrometry (LC–MS/MS), which provides superior sensitivity and selectivity [31,32] and is well suited for less volatile, thermally labile OPEs in soil matrices. Furthermore, the multiple reaction monitoring (MRM) mode of LC–MS/MS effectively minimizes matrix interferences, markedly enhancing both selectivity and detectability. Consequently, LC–MS/MS has become the method of choice for trace-level OPE determination in complex environmental samples.

Current soil analysis for organophosphate esters is hindered by low pretreatment efficiency, variable recoveries, and limited method applicability. It is therefore imperative to establish a reliable multi-residue method capable of simultaneously determining OPEs in soil. In this study, we selected 25 representative novel OPEs—including 10 alkyl, 3 halogenated, and 12 aryl compounds—as target analytes. Using accelerated solvent extraction as the core technique, we systematically optimized solvent type and ratio to maximize recoveries for all targets. To reduce co-extracted interferences (e.g., lipids, humic acids, and pigments), we evaluated single and combined adsorbents (PSA, C18, and GCB) to identify the most effective cleanup strategy for minimizing matrix effects. We further optimized LC-MS/MS parameters and established the separation and detection conditions for the 25 OPEs, followed by full method validation covering linear range, limits of detection (LODs), precision (RSD), and spike recoveries. The resulting method is sensitive, efficient, and accurate for simultaneous OPE determination in soil, offering reliable technical support for environmental monitoring and risk assessment.

## 2. Materials and Methods

### 2.1. Samples

A total of 15 agricultural soil samples were collected from a region in Ningbo, Zhejiang Province, between October and November 2025, including 9 paddy field soils and 6 vegetable field soils, representing the major cereal- and cash-crop cultivation areas in the study region, respectively.

Visible surface debris was removed prior to sampling. At each site, three to five surface soil subsamples (0–20 cm depth) were collected following a five-point (plum-blossom) sampling pattern, thoroughly mixed, and sealed in amber glass bottles. Samples were transported in coolers with ice packs to maintain low temperature. Blank samples were prepared by baking field soil at high temperature. Samples were freeze-dried within 48 h of collection. After delivery to the lab, they were stored at −20 °C in amber glass jars with Teflon-lined caps. The maximum storage time before extraction was 14 days.

### 2.2. Instruments, Reagents and Materials

Instruments used included an accelerated solvent extraction system: Speed Extractor E-916 (Büchi Labortechnik AG, Flawil, Switzerland); liquid chromatography–tandem mass spectrometry system: SCIEX ExionLC™ coupled with Triple Quad 3500 (SCIEX, Marlborough, MA, USA); rotary evaporator: Hei-VAP Precision (Heidolph, Schwabach, Bavaria, Germany); vortex mixer: VORTEX GENIUS 3 (IKA, Staufen, Baden-Württemberg, Germany); and analytical column: ZORBAX Eclipse Plus C18 (150 mm × 2.1 mm, 3.5 μm) (Agilent, Santa Clara, CA, USA).

Methanol and acetonitrile (HPLC grade, Thermo Fisher, Waltham, MA, USA); distilled water (4.5 L, Watsons, Guangzhou, China); ammonium acetate (97%, Alfa Aesar, Ward Hill, MA, USA); disposable organic syringe filters (nylon, 13 mm, 0.22 μm) (Shanghai Anpel Laboratory Technologies Co., Ltd., Shanghai, China); anhydrous sodium sulfate (analytical reagent grade, Sinopharm Chemical Reagent Co., Ltd., Shanghai, China); and diatomaceous earth (10–20 mesh, Thermo Fisher, USA) were used.

Twenty-five OPE standards (9 alkyl, 4 halogenated, and 12 aryl compounds) were obtained from suppliers including Anpel Trace (Shanghai, China), Dr. Ehrenstorfer, TMstandard (Changzhou, China), CATO (Guangzhou, China), MACKLIN (Shanghai, China), and ZZBIO (Shanghai, China). The full list comprises: TMP, TEP, TZ3P, TPrP, TiBP, TnBP, TCPP, TPP, TXP, TOP, EHDPP, TCEP, TBEP, TPeP, m-TCP, p-TCP, o-TCP, TDCP, TPPO, TBPP, THP, IPPP, BDP, CDP, and TiPPP. Two isotopically labeled internal standards (TPP-d15 and TMP-d9) were purchased from Anpel Trace for quantitative analysis. All analytical standards had purity >98%.

### 2.3. Samples Pretreatment

An air-dried soil sample (10.0 g, weighed to 0.01 g) was transferred into an ASE cell. After removing roots, stones, and other debris, an appropriate amount of diatomaceous earth was added, and the mixture was thoroughly ground to a granular consistency. The sample was then spiked with 0.1 mL of mixed standard solution (1 μg/mL) and 50 μL of internal standard solution (10 ng/mL), mixed, and allowed to equilibrate for 30 min.

Extraction was performed with dichloromethane–methanol (1:1, *v*/*v*) under the following ASE conditions: pressure 100 bar, temperature 100 °C, heat-up time 5 min, static time 5 min, flush volume 60%, and two cycles. The extract was collected and concentrated to 2–3 mL by rotary evaporation. If visible water was present, anhydrous sodium sulfate was added, and the mixture was left to stand for 15 min for dehydration. The supernatant was then transferred, and the original container was rinsed with a small volume of methanol; all rinsates were combined with the extract for subsequent cleanup.

The combined extract was transferred to a 5-mL volumetric flask. The container was rinsed with methanol, and the rinsings were added to the flask, followed by the internal standard solution (10 ng/mL). The volume was made up to the mark with methanol. A 1-mL aliquot of this solution was taken, mixed with 300 mg of PSA and 50 mg of C18, and shaken for 3 min. After thorough settling, the supernatant was collected for instrumental analysis.

### 2.4. Instrumental Analysis

LC-MS/MS analysis was performed on a SCIEX Triple Quad 3500 system equipped with a ZORBAX Eclipse Plus C18 column (150 mm × 2.1 mm, 3.5 µm). The mobile phase consisted of 0.1% formic acid in water (phase A) and methanol (phase B), with a column temperature of 35 °C and an injection volume of 2 µL. The gradient program was: 0–5 min, 70% B; 5–17 min, 70 → 74% B; 17–18 min, 74 → 100% B; 18–22 min, 100% B; and 22–25 min, 70% B. The flow rate was 0.4 mL/min.

**Figure 1:**
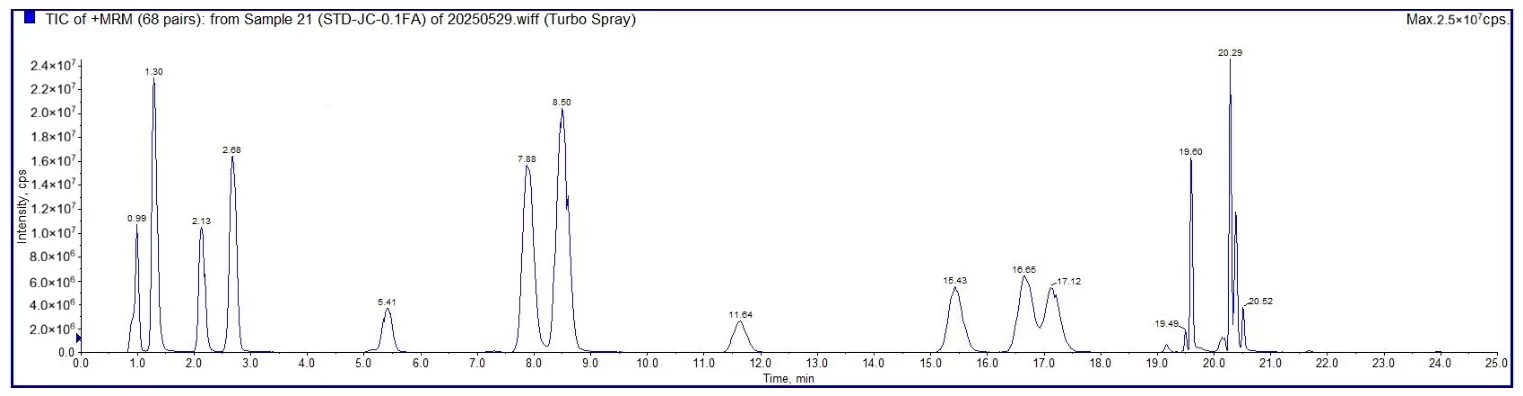
The MRM chromatograms of standard solution.

Mass detection was carried out in positive-ion electrospray ionization (ESI) with multiple reaction monitoring (MRM). The optimized source parameters were: electrospray voltage 5500 V, source temperature 550 °C, curtain gas 40 kPa, nebulizer gas 55 kPa, and auxiliary gas 60 kPa. The precursor/product ion transitions, collision energies (CE), decluster potential (DP), retention times (RT), and CAS numbers for each target compound are summarized in Table 1 and Table 2. The MRM chromatograms of standard solution are shown in Figure 1.

### 2.5. Quality Control and Assurance

To minimize background contamination, plastic and rubber materials were avoided throughout sampling and analysis. All glassware was rinsed three times with ultrapure water and methanol prior to use. Standards and internal standards were stored at −20 °C in the dark as per the manufacturer’s instructions and equilibrated to room temperature before use. Anhydrous sodium sulfate was baked at 650 °C for 4 h to remove moisture and organic impurities, and diatomaceous earth was heated at 400 °C for 4 h, then cooled and kept in a sealed dry container. During sample pretreatment, two procedural blanks were included per batch of ten samples to monitor any contamination introduced during the process.

## 3. Results and Discussion

### 3.1. Optimization of Extraction Solvent

The 25 target OPEs in this study span a broad range of physicochemical properties, from polar, low-molecular-weight trimethyl phosphate (TMP) to highly nonpolar trioctyl phosphate (TOP), with log Kow values covering a wide spectrum. Given the complexity of the soil matrix and the varying adsorption affinities of organic matter and minerals toward different OPEs, selecting a suitable extraction solvent is crucial for maximizing recoveries while minimizing co-extracted interferences. Mixed solvent systems offer distinct advantages over single solvents (e.g., hexane, acetone, dichloromethane, or methanol) by combining complementary polarities and solubilities, thereby accommodating diverse OPEs and reducing adsorption losses onto soil components.

**Figure 2:**
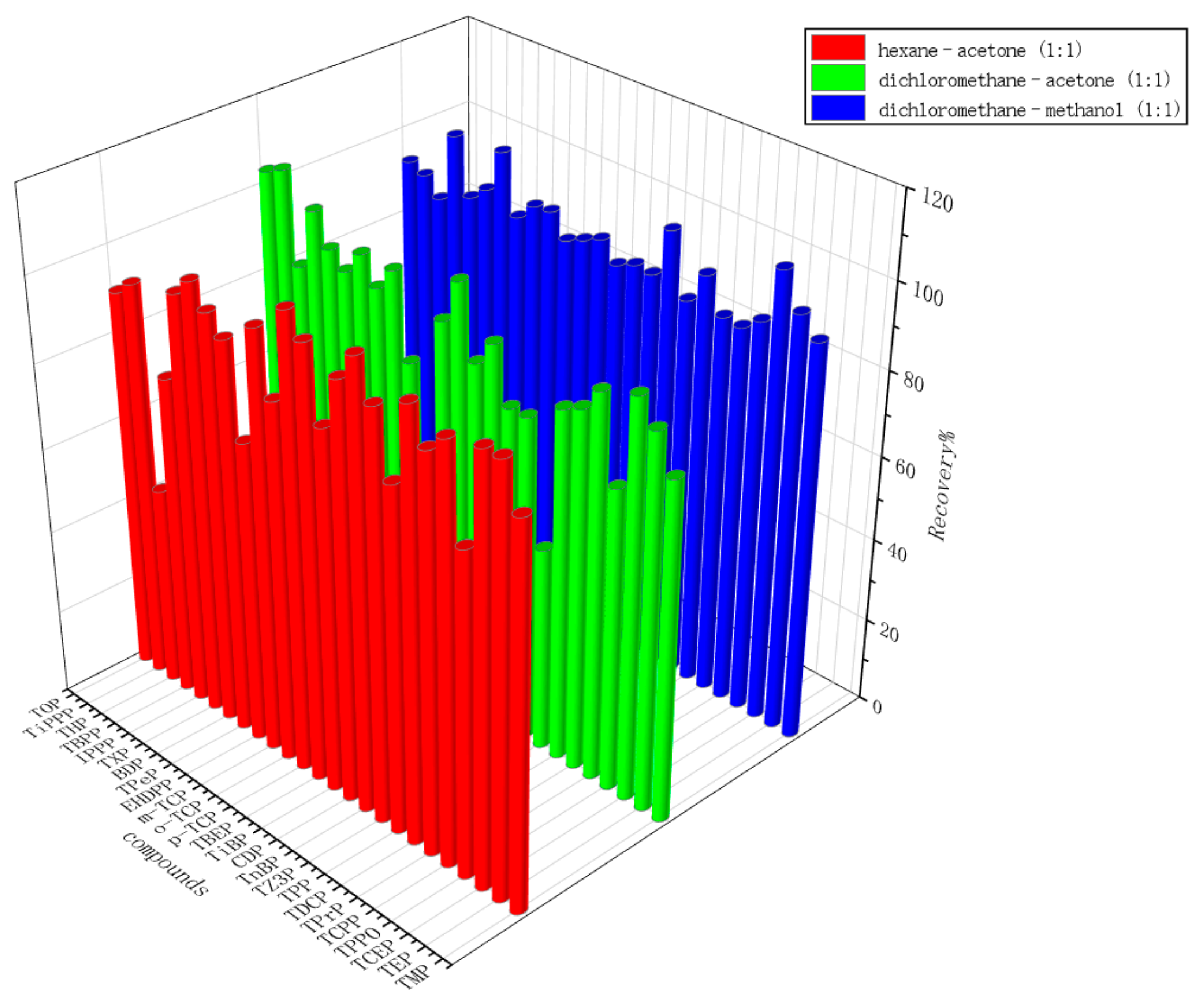
Extraction efficiencies of the three solvents.

We compared three binary solvent mixtures—hexane–acetone (1:1), dichloromethane–acetone (1:1), and dichloromethane–methanol (1:1)—each tested in triplicate with procedural blanks (Figure 2). Hexane–acetone gave recoveries of 76.4–107% for 24 compounds, but only 47.7% for THP, likely due to its strong hydrophobicity (high log Kow) and steric hindrance from three hexyl groups, which limit solubility and release from organic matter. Dichloromethane–acetone afforded recoveries of 48.9–106% for most OPEs, yet failed for TPP and o-TCP (<70%), presumably because these aryl compounds engage in strong π–π stacking with aromatic soil constituents that this system cannot disrupt. In contrast, dichloromethane–methanol (1:1) yielded recoveries of 87.6–105% for all 25 compounds, meeting the 70–120% acceptance criteria for trace analysis. The moderately polar to polar nature of this mixture effectively dissolves OPEs of all polarities, and Dichloromethane effectively disrupts soil organic matter binding, while methanol—as a protic solvent—can break hydrogen bonds between analytes and soil organic matter, enhancing extraction efficiency.

Based on these results, dichloromethane–methanol (1:1, *v*/*v*) was selected as the optimal extraction solvent for the ASE of the 25 OPEs in soil samples.

### 3.2. Optimization of Sorbents

After accelerated solvent extraction of soil samples, the crude extracts contain abundant co-extracted interferences—lipids, humic acids, pigments, and sulfides—that can severely compromise LC-MS/MS sensitivity, accuracy, and column lifetime. Thus, an effective cleanup step is indispensable.

Two primary cleanup strategies exist: solid-phase extraction (SPE) cartridges and dispersive SPE (d-SPE). Previous studies on OPEs in air, dust, and biota have often employed neutral silica or Florisil columns with ethyl acetate as eluent [33]. However, our soil matrices are richer in organic matter and pigments. When we applied ethyl acetate elution through either silica or Florisil, substantial co-extracted organic matter was co-eluted, yielding a dark yellowish-brown, viscous extract that formed a gel-like residue during nitrogen concentration, could not be dried completely, and remained turbid upon reconstitution—rendering instrumental analysis and quantitation unreliable.

We therefore adopted d-SPE cleanup, which simply requires adding sorbent to the concentrated extract, vortex-mixing, and centrifuging—eliminating the tedious conditioning, washing, and elution steps of cartridges. Moreover, SPE cartridges are typically polypropylene and may release plasticizers or antioxidants that introduce OPE background contamination; d-SPE avoids this risk. Its cleanup capacity is also readily adjustable by varying sorbent amounts, accommodating large-volume extracts without the capacity constraints of SPE cartridges.

Sorbent type critically influences both cleanup efficiency and analyte recovery. Commonly used sorbents—PSA (primary secondary amine), C18, and GCB (graphitized carbon black)—were compared, with PSA and C18 previously reported as suitable for OPEs [34,35]. We evaluated PSA (50 mg), C18 (50 mg), GCB (50 mg), and a mixture of PSA 25 mg + C18 25 mg using blank soil extracts spiked with 0.1 mL of mixed standard (1 μg/mL), adjusted to 1 mL, vortexed for 3 min, centrifuged, and filtered through 0.22 μm membranes before LC-MS/MS analysis.

**Figure 3:**
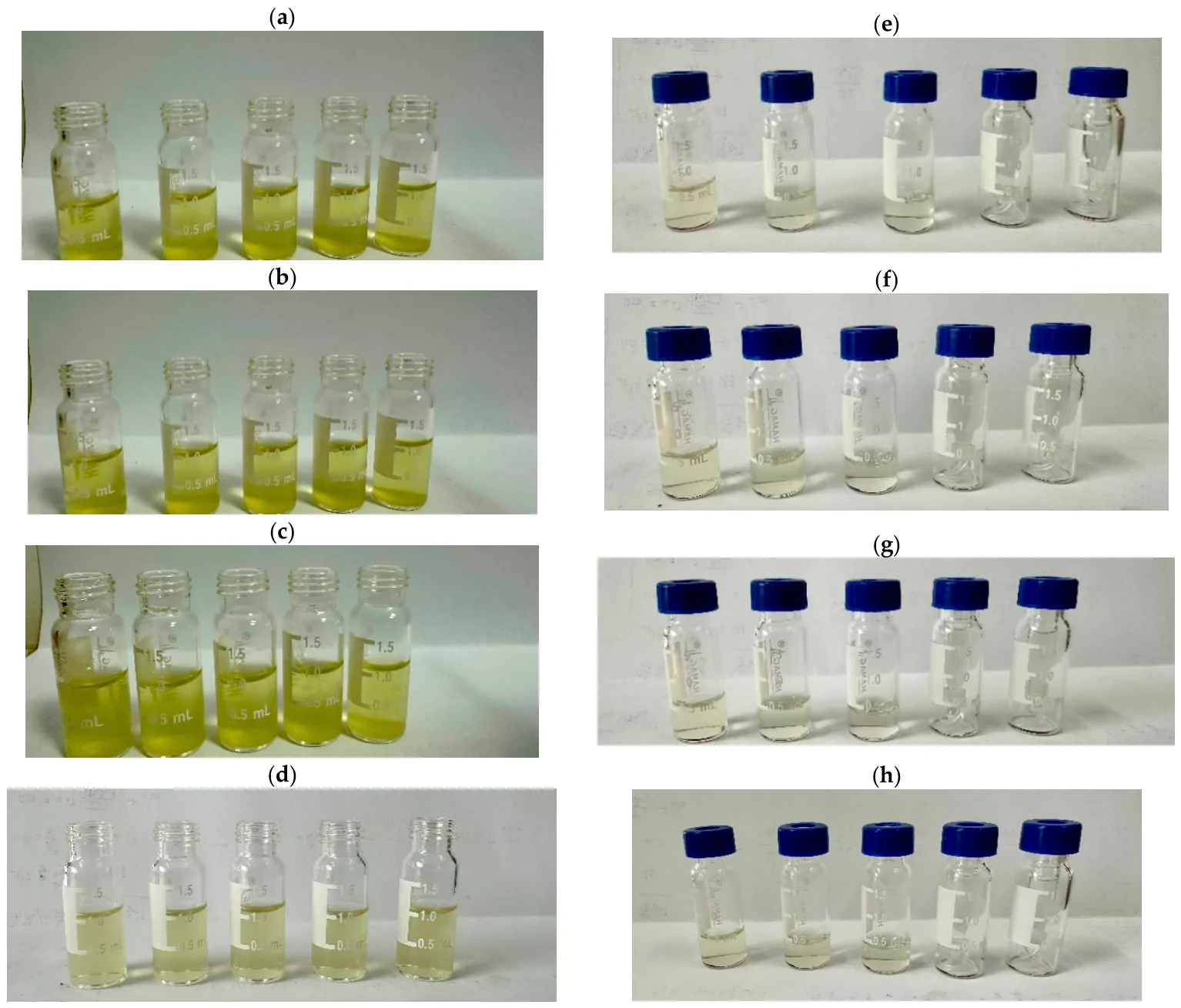
Comparison of pre- and post-d-SPE clean-up. The visual appearance of the sample matrices: (**a**–**d**) without sorbent, (**e**) with PSA, (**f**) with C18, (**g**) with PSA + C18, and (**h**) with GCB.

Cleanup effectiveness was visually evident: the untreated solution was dark yellow and turbid (abundant pigments and organics), whereas PSA or C18 produced a light yellow, clear solution, and GCB gave a colorless, transparent solution (Figure 3), confirming efficient removal of pigments and other interferents.

**Figure 4:**
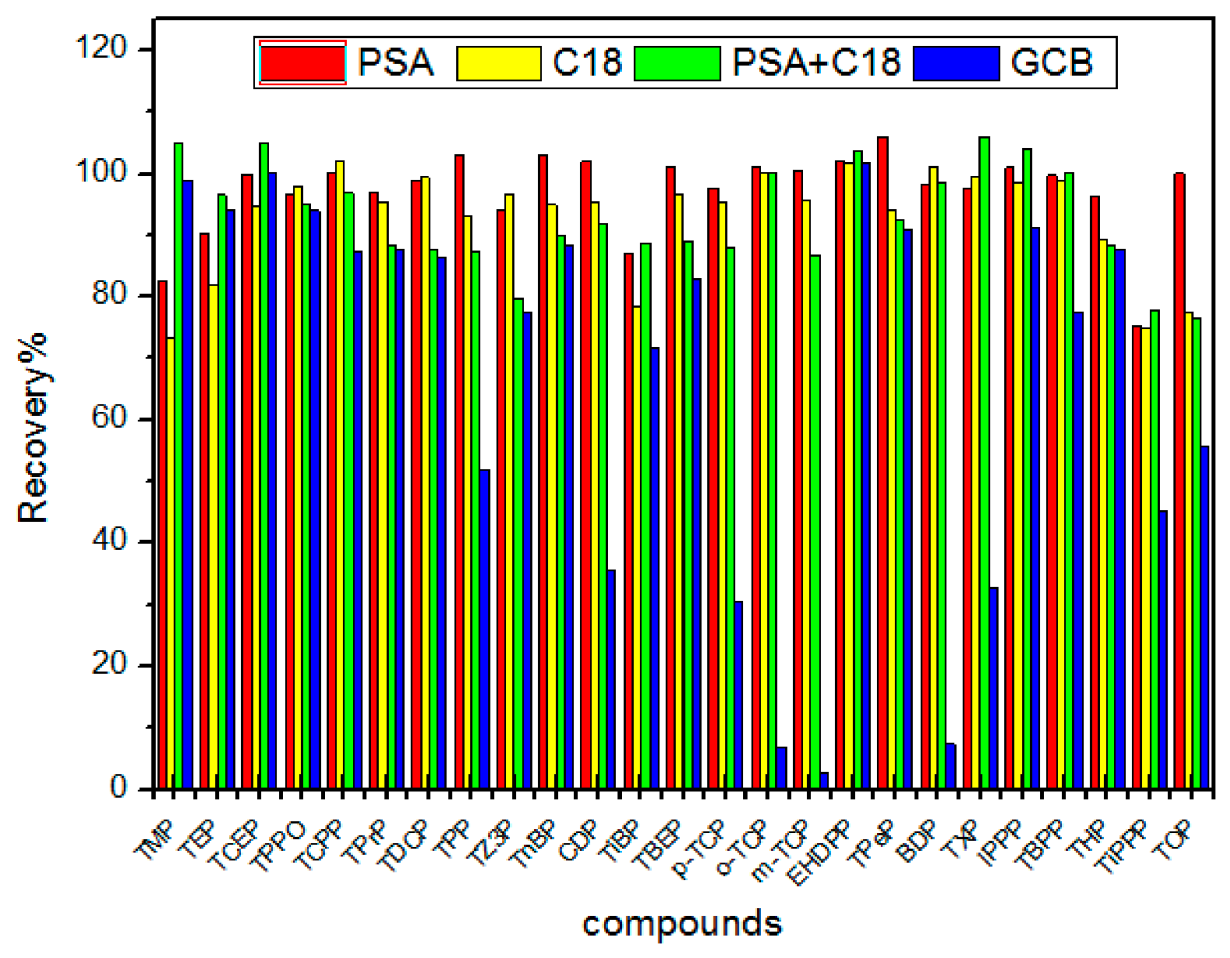
Recovery of target OPEs with different adsorbents.

Recoveries with C18, PSA, and C18/PSA were comparable: 75.2–103%, 73.2–102%, and 76.5–105%, respectively (Figure 4). In contrast, GCB caused poor recoveries for some OPEs, with m-TCP and BDP falling below 10%. This is attributable to the highly ordered hexagonal planar structure of GCB, which exerts strong π–π stacking interactions with planar, benzene-ring-containing molecules, leading to irreversible adsorption of aryl OPEs and preventing desorption.

Consequently, the combination of C18 and PSA was chosen as the optimal sorbent mixture.

### 3.3. Selection of Sorbent Dosage

During d-SPE cleanup, sorbents can adsorb not only matrix interferences but also some target OPEs via non-specific interactions, potentially reducing analyte recoveries. Insufficient sorbent leaves impurities incompletely removed, causing pronounced matrix effects, while excessive sorbent may lead to irreversible adsorption losses, particularly for aryl OPEs. Thus, optimizing sorbent dosage is critical for balancing cleanup efficiency and satisfactory recoveries.

We employed a standard-addition approach to determine the optimal dosage range for each sorbent. Briefly, 1 mL of blank soil extract was spiked with a mixed standard of the 25 OPEs (20 ng/mL) and treated with varying amounts of PSA, C18, or GCB. After vortexing (3 min), centrifugation, and filtration (0.22 μm), the supernatants were analyzed by LC-MS/MS. Tested PSA dosages were 50, 100, 150, 200, 250, 300, 400, and 500 mg; C18 dosages were 50, 100, 150, 200, and 250 mg; GCB dosages were 0.25, 5, 10, 20, 30, 40, and 50 mg.

**Figure 5:**
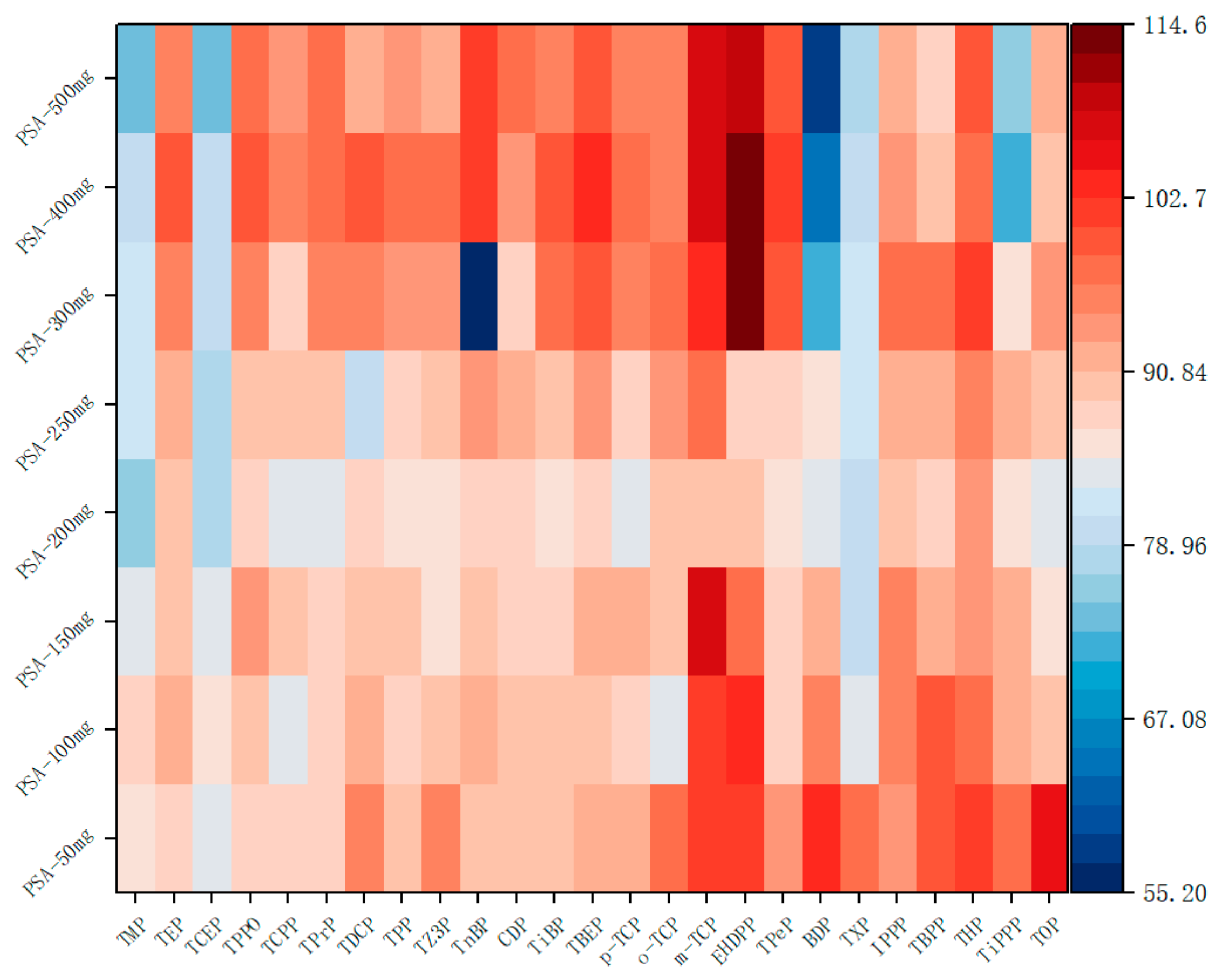
Effect of PSA concentration on the recovery of target OPEs.

PSA dosage (Figure 5): Recoveries of all 25 OPEs remained within 73.7–114.5% across the 50–500 mg range, indicating negligible non-specific adsorption by PSA. Given the greater complexity of real soil extracts, a PSA dosage of 100–500 mg is recommended to effectively suppress matrix effects, with the exact amount adjustable according to sample organic matter content.

**Figure 6:**
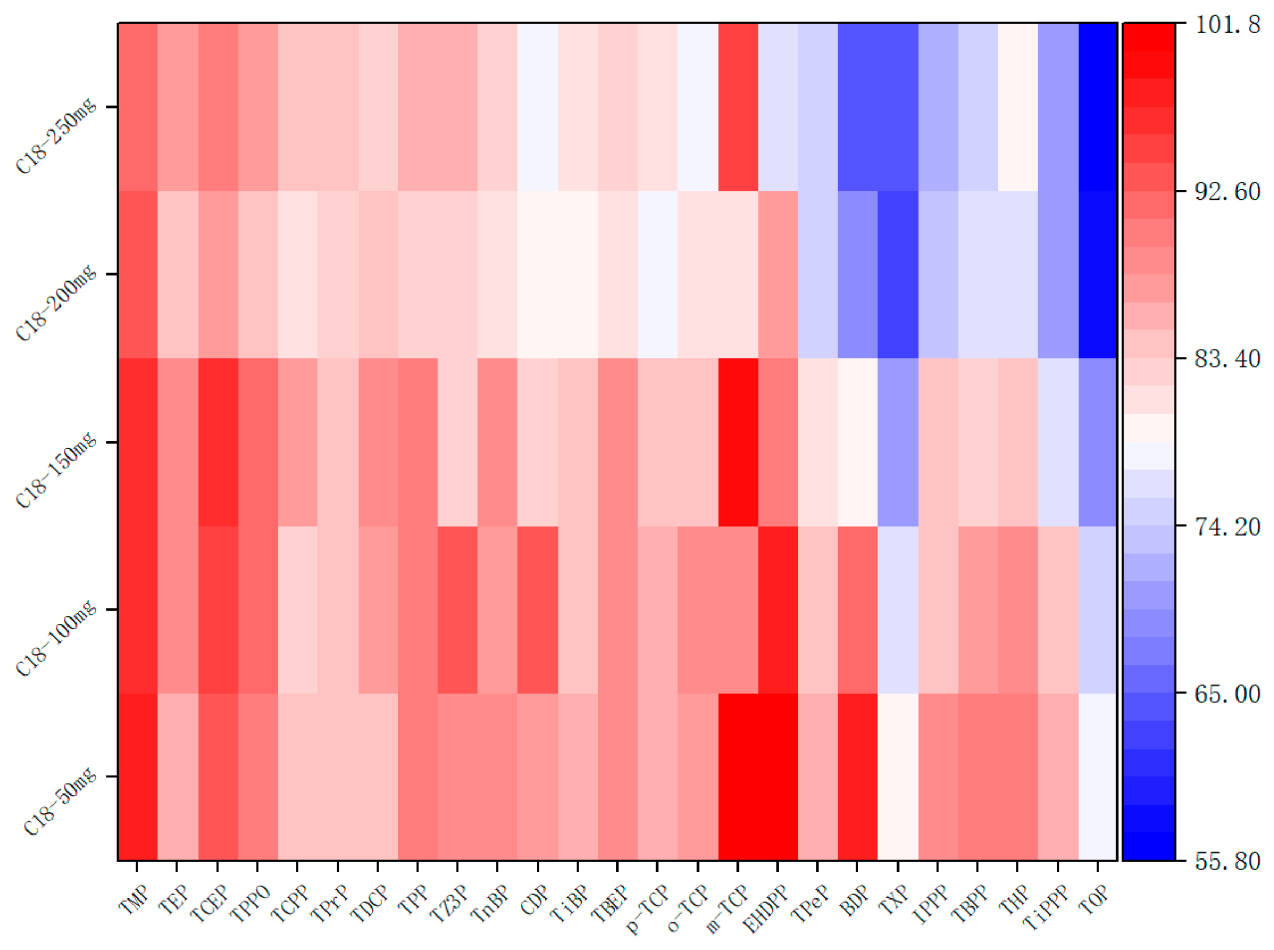
Effect of C18 concentration on the recovery of target OPEs.

C18 dosage (Figure 6): Recoveries slightly decreased as C18 increased from 50 to 250 mg, likely because higher amounts retained certain OPEs (e.g., EHDPP and TOP). Recoveries with 50 mg and 100 mg C18 were comparable (78.8–101.8% vs. 74.3–97.3%); therefore, 50 mg was chosen as the optimal C18 dosage.

**Figure 7:**
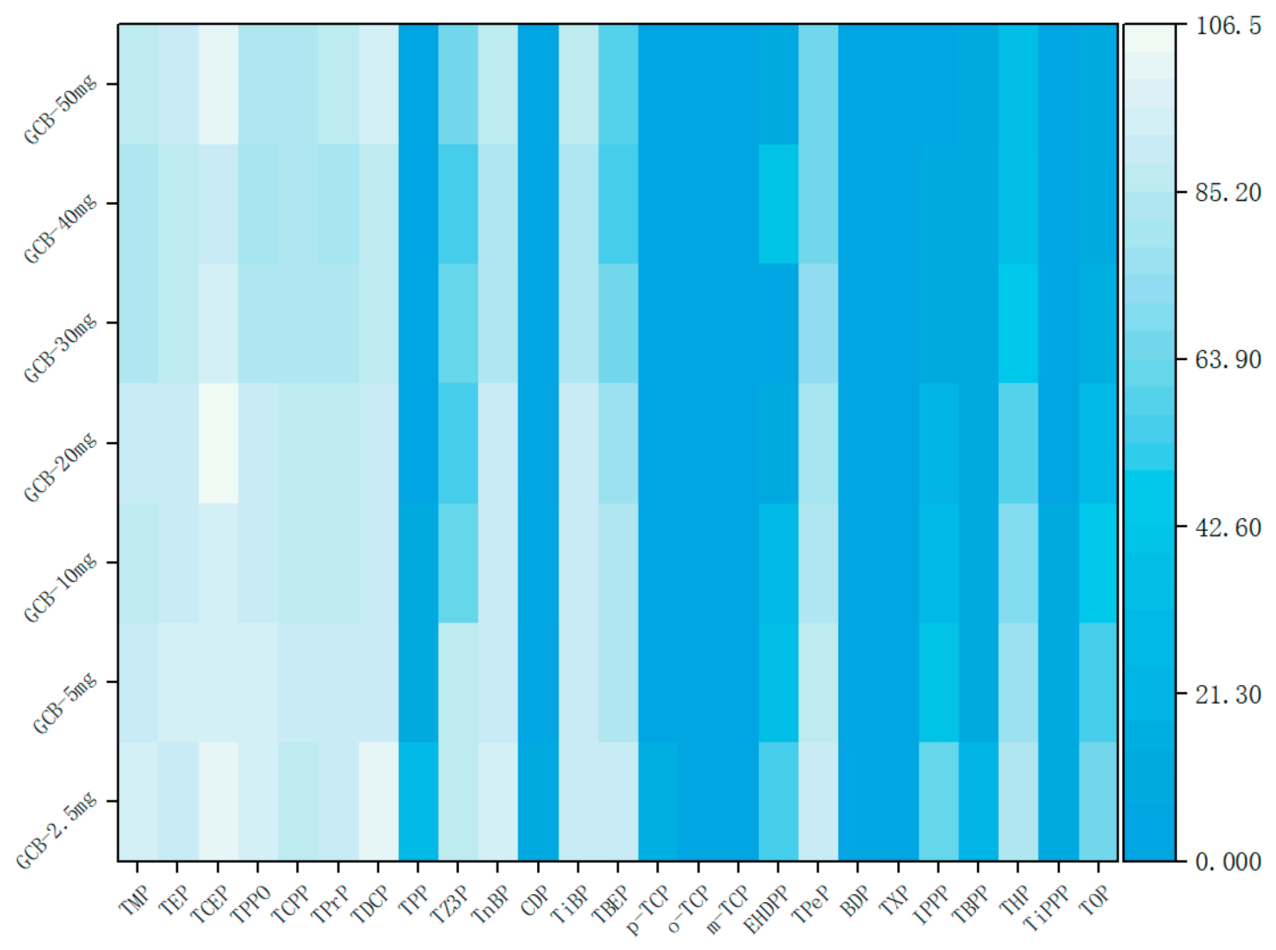
Effect of GCB concentration on the recovery of target OPEs.

GCB dosage (Figure 7): GCB showed strong adsorption affinity for most OPEs. As the GCB amount rose from 0.25 to 50 mg, recoveries of aryl OPEs—including TPP, CDP, TCP, BDP, TXP, TBPP, and IPPP—fell sharply, dropping below 50% at ≥2.5 mg and approaching total loss at higher levels. In contrast, alkyl OPEs (e.g., TMP, TEP, TCEP, TPPO, TCPP, TPrP, TDCP) maintained >80% recovery even at 50 mg GCB. This differential behavior is attributed to the strong, irreversible π–π stacking interactions between the graphitized planar surface of GCB and benzene-ring-containing molecules, which prevent desorption by organic solvents. Despite GCB’s excellent pigment removal, its severe retention of aryl OPEs—nearly half of our targets—renders it unsuitable for multi-class OPE analysis.

Based on these findings, the final cleanup combination was set at 50 mg C18 + 300 mg PSA.

### 3.4. Method Validation

A series of working standard solutions at 1, 5, 10, 20, 50, and 100 ng/mL was prepared by serial dilution of the 1 μg/mL mixed stock solution in methanol. To each standard, 50 μL of isotopically labeled internal standard mixture (containing TPP-d15 and TMP-d9) was added, yielding a fixed internal standard concentration of 50 ng/mL in the final injection solution. After thorough mixing, the solutions were analyzed by LC-MS/MS. Calibration curves were constructed by plotting the peak-area ratio of each target compound to its corresponding internal standard (y) against the mass concentration of the target compound (x, ng/mL), and linear regression was performed using the least-squares method to obtain the regression equations and correlation coefficients.

The regression equations, correlation coefficients (R^2^), and linear ranges for all 25 OPEs are summarized in Table 3. All compounds showed excellent linearity over the tested ranges, with R^2^ > 0.994 for all and >0.996 for most, meeting the requirements for trace-level OPE determination in soil.

For compounds detectable in procedural blanks, the measured concentrations from seven replicate analyses were used to calculate the standard deviation (SD), and the method detection limit (MDL) was determined as three times the SD. For compounds not detected in blanks, the concentration corresponding to a signal-to-noise ratio of 3 (S/N = 3) was employed. In this study, the MDL and LOQ were determined by analysing seven replicates of soil samples spiked with 5.0 ng/g OPEs (S/N = 3) following the full ASE–d-SPE–LC-MS/MS workflow. MDL and LOQ were calculated as 3 × SD and 10 × SD of the replicate results, respectively.

The MDL was calculated according to the formula:

MDL = t (n − 1, 0.99) × SD(1)

where *n* is the number of replicate determinations, *t* is Student’s t-value at the 99% confidence level with *n* − 1 degrees of freedom, and *SD* is the standard deviation of the measured concentrations from the *n* replicates.

The final extract volume for the method is 1.0 mL. The soil dry weight is 10.0 g. The conversion factor is: Sample concentration (ng/g) = [Concentration in extract (ng/mL) × 1.0 mL]/10.0 g = Concentration in extract (ng/mL)/10.

The MDLs were calculated for OPEs in the range of 0.01–1.08 ng/g. To prove the feasibility of this method, a comparison was conducted for extracting OPEs in soil samples using the developed ASE method and previously reported methods [36,37,38]. The published methods primarily involved ultrasonic and/or oscillation extraction–SPE, microwave-assisted extraction–SPE and Soxhlet extraction–SPE. As shown in Table 3, the recoveries in this work were better than those obtained by microwave-assisted extraction–SPE, and comparable to those achieved by Soxhlet extraction–SPE and ultrasonic extraction–SPE. The MDLs of OPEs in the optimized method were lower than those obtained by microwave-assisted extraction–SPE, Soxhlet extraction–SPE and ultrasonic extraction. In general, the recoveries and MDLs of all target compounds in this work were comparable to those of other published methods.

In this study, matrix effects (ME) were evaluated by comparing the signal responses of target compounds spiked into the post-extraction matrix with those in pure solvent at equivalent concentrations, following previously established methodology [39].

The ME was determined based on the following equation:

ME = [(B − A)/C] × 100%(2)

where *B* is the peak area of an analyte spiked into a post-extraction blank matrix extract. *A* is the blank matrix response, and *C* is the response in pure solvent.

According to the value of ME, the level of ME was classified as follows [39]: “low ME” (ME of 80–120%), “moderate ME” (ME of 40–80% or 120–150%), and “strong ME” (ME < 40% or ME > 150%). As shown in Table 3, MEs of the OPEs were moderate.

### 3.5. Accuracy and Precision

Method accuracy and precision were evaluated through spike recovery tests using farmland soil samples, which were confirmed (by preliminary analysis) to be free of target OPEs or to contain them only below detection limits. Recovery experiments were performed at three spiking levels—low (1 ng/g), medium (50 ng/g), and high (100 ng/g)—with six replicates (*n* = 6) per level. For each compound, the mean recovery and relative standard deviation (RSD) were calculated, and the results are summarized in Table 4.

At the three levels, recoveries of the 25 OPEs ranged from 70.6% to 107%, 76.2% to 109%, and 77.4% to 111%, respectively, with all RSDs below 12%. All recoveries fell within the acceptable 70–120% range, with the majority concentrated between 80% and 110%. Slightly lower recoveries (approximately 70–80%) were observed for some volatile alkyl OPEs (e.g., TMP and TCEP), likely due to evaporative losses during concentration. Overall, the method exhibits good accuracy and reproducibility, sufficient for trace-level soil contaminant analysis.

For samples containing endogenous target compounds, the recovery should be corrected for background concentration using the following equation:

Recovery (%) = [(C_1_ − C_0_)/C] × 100% (3)

where *C*_1_ is the analyte concentration in the spiked sample, *C*_0_ is the native concentration in the unspiked soil, and *C* is the theoretical spiked concentration (from the standard solution).

### 3.6. Application to Field Soil Samples

To assess the feasibility and reliability of the proposed method for environmental sample analysis, we applied the newly developed ASE-d-SPE-LC-MS/MS method to determine the concentrations of 25 target OPEs in agricultural soils from a region in Ningbo, Zhejiang Province, China. Surface soil samples (0–20 cm) were collected from multiple plots within the study area. Upon arrival at the laboratory, the samples were freeze-dried, ground, and sieved through a 100-mesh sieve, and subsequently analysed according to the pretreatment protocol and instrumental conditions detailed in this study.

Soil contamination by organophosphate esters (OPEs) has been widely reported across China. For instance, ΣOPE concentrations in urban soils from Guangzhou ranged from 41.0 to 1370 ng/g [40], and in surface soils from Chongqing from 10.1 to 315 ng/g [8]. Moreover, detectable levels of OPEs have also been found in soils from other Chinese cities, such as Shenyang [9] and Fuzhou [41], indicating the widespread occurrence of these compounds in diverse urban environments.

The detection results of the 25 target OPEs in the actual soil samples are presented in Table 5. The results indicated that the concentrations of most target OPEs in the soil samples from this region were below the method detection limits (MDLs), which is closely related to factors such as land-use types, distribution of pollution sources, and soil physicochemical properties. The physicochemical properties of the actual soil samples in this region were as follows: pH ranged from 5.28 to 8.23, organic matter content ranged from 16.6 to 30.1 g/kg, Total organic carbon (TOC) ranged from 9.6to 17.5 g/kg, and the soil types included loamy clay, silty clay, and clay loam.

Among the detectable compounds, tris(2-chloroisopropyl) phosphate (TCPP) exhibited the highest concentrations, emerging as the predominant OPE congener in these soils. This observation is consistent with TCPP’s extensive industrial use and environmental persistence. As a halogenated organophosphate ester flame retardant, TCPP is widely applied in polyurethane foams, building insulation, furniture, textiles, and other sectors, owing to its high flame-retardant efficiency, thermal stability, low volatility, and good polymer compatibility. Zhejiang Province, a major manufacturing hub in China, hosts highly developed footwear, furniture, construction, and textile industries, all of which consume large quantities of TCPP-containing raw materials. Throughout the product lifecycle—from manufacturing and use to disposal—TCPP can be released into the environment.

Toxicological and ecotoxicological studies have demonstrated that various OPEs exert neurotoxic, carcinogenic, and endocrine-disrupting effects on organisms [42]. Chlorinated OPEs exhibit high environmental persistence and bioaccumulation potential [43]. TCEP has been classified as a Category 2 carcinogen by the European Union [5], while TDCP, TCPP, and TBEP have been reported to pose potential carcinogenic risks [44]. TnBP can interfere with the endocrine system and impair reproductive function and neurodevelopment [45]. Furthermore, TCPP has been shown to exert neurotoxicity in fish, affecting the neural development of zebrafish embryos [46]. TnBP can accumulate in the intestinal tract of earthworms, disrupting intestinal barrier integrity, impairing nutrient absorption, disturbing gut microbiota homeostasis, and inducing intestinal toxicity [47].

Soils serve as a major environmental sink for various persistent organic pollutants. The extensive use of plastic films in greenhouses [48] may further increase the risk of OPE contamination in agricultural soils. Once introduced into soil, OPEs can be taken up and accumulated by plant roots, subsequently entering the food chain and posing a potential threat to human health. Consequently, heightened attention should be directed toward the occurrence, source identification, and potential ecological and health risks of OPEs in soil.

## 4. Conclusions

In this study, a rapid and efficient pretreatment method was developed for the extraction of 25 organophosphate esters (OPEs) from soil samples. Several key parameters affecting extraction efficiency were systematically optimized. The optimal procedure employed accelerated solvent extraction (ASE) with dichloromethane–methanol (1:1, *v*/*v*) as the extraction solvent, followed by dispersive solid-phase cleanup using a mixed sorbent comprising PSA and C18. When coupled with highly selective LC-MS/MS detection, the method provided satisfactory recoveries and low method detection limits (MDLs) for all target compounds. The developed method was successfully applied to the analysis of OPEs in actual soil samples, with detectable concentrations ranging from not detected (ND) to 11.4 ng/g.

The pretreatment workflow is operationally simple and convenient. Through optimization of extraction and cleanup conditions, background contamination was effectively eliminated, and the method’s resistance to matrix interference was substantially enhanced. The method enables simultaneous determination of 25 OPEs, offering excellent recoveries (70.6–111%) and low detection limits (0.01–1.08 ng/g). Validation with real soil samples demonstrated that the method exhibits good applicability to soils with varying matrix compositions and fully meets the requirements for trace-level OPE determination, making it particularly suitable for large-scale environmental monitoring programs. Therefore, the established method holds great promise for broad application in the simultaneous analysis of 25 OPEs.

## Figures and Tables

**Table 1:** Names and basic physicochemical parameters of the target OPEs.

Compounds	Molecular Formula	CAS	Abbreviation	Molecular Weight
**Alkyl OPEs**				
Trimethyl phosphate	C_3_H_9_0_4_P	512-56-1	TMP	140.08
Triethyl phosphate	C_6_H_15_O_4_P	78-40-0	TEP	182.15
Tris(2-butoxyethyl) phosphate	C_18_H_39_O_7_P	78-51-3	TBEP	398.5
Tributyl Phosphate	C_12_H_27_O_4_P	126-73-8	TnBP	266.31
Tipentyi	C_15_H_33_O_4_P	2528-38-3	TPeP	308.39
Tri-propyl phosphate	C_9_H_21_O_4_P	513-08-6	TPrP	224.23
Trihexyl	C_18_H_39_O_4_P	2528-39-4	THP	350.47
Tri-isobutyl phosphate	C_12_H_27_O_4_P	126-71-6	TiBP	266.31
Trioctyl	C_24_H_51_0_4_P	1806-54-8	TOP	434.62
**Halogenated OPEs**				
Tris(2-chloroethyl) phosphate	C_6_H_12_Cl_3_O_4_P	115-96-8	TCEP	285.5
Tris(1,3-dichloro-2-propyl) phosphate	C_9_H_15_Cl_6_O_4_P	13674-87-8	TDCP	430.9
Tris(2,3-dibromopropyl) phosphate	C_9_H_15_Br_6_O_4_P	126-72-7	TZ3P	697.61
Tris(2-chloroisopropyl) phosphate	C_9_H_18_Cl_3_O_4_P	13674-84-5	TCPP	327.56
**Aryl OPEs**				
Triphenylphosphine oxide	C_18_H_15_OP	791-28-6	TPPO	278.28
Triphenyl phosphate	C_18_H_15_O_4_P	115-86-6	TPP	326.3
Diphenyl p-tolyl phosphate	C_19_H_17_O_4_P	26444-49-5	CDP	340.3
Tri-p-cresyl phosphate	C_21_H_21_O_4_P	1330-78-5	m-TCP	368.4
2-Ethylhexyl diphenyl phosphate	C_20_H_27_O_4_P	1241-94-7	EHDPP	326.4
Bis(triphenyiphosphate)	C_36_H_30_O_8_P_2_	5946-33-8	BDP	628.56
Trixylylphosphate	C_24_H_27_O_4_P	25155-23-1	TXP	410.44
Tri(4-isopropylphenyl) phosphate	C_27_H_33_O_4_P	68937-41-7	IPPP	436.53
Tris(4-tert-butylphenyl) phosphate	C_30_H_39_O_4_P	78-33-1	TBPP	494.6
Tri-o-cresyl phosphate	C_21_H_21_O_4_P	78-30-8	o-TCP	352.38
Tri-p-cresyl phosphate	C_21_H_21_O_4_P	78-32-0	p-TCP	352.38
Tris(isopropylphenyl) phosphate	C_27_H_33_O_4_P	64532-95-2	TiPPP	452.5

**Table 2:** Mass spectrometric parameters of OPEs.

NO.	Compound	Retention Time (min)	Ion Pairs (*m*/*z*)	Decluster Potential/V	Collision Energy/V
1	TMP	1.00	141.0/109.1, 141.0/79.0	70	23
2	TEP	1.26	183.1/98.9, 183.1/155.0	50	25
3	TCEP	1.36	285.0/98.7, 285.0/63.1	71	34
4	TPPO	2.04	279.0/201.1, 279.0/173.0	122	38
5	TCPP	2.50	327.0/98.8, 327.0/251.0	60	40
6	TPrP	2.55	225.1/99.0, 225.1/141	50	30
7	TDCP	4.67	431.1/99.1, 431.1/209	83	48
8	TPP	4.90	326.9/77.0, 326.9/219	120	62
9	TZ3P	6.53	698.6/99, 698.6/298.9	86	70
10	TnBP	7.68	267.0/99.0, 267.0/211.0	54	30
11	CDP	7.30	340.8/152.1, 340.8/91.0	120	45
12	TiBP	7.14	267.1/99.0, 267.1/211.1	55	30
13	p-TCP	7.90	369.1/91.1, 369.1/165.1	120	60
14	TBEP	10.36	399.1/299.2, 399.1/199.0	85	20
15	o-TCP	15.09	369.1/91.1, 369.1/165.1	120	60
16	m-TCP	15.46	369.1/91.1, 369.1/165.1	125	65
17	EHDPP	19.24	363.0/251.0, 363.0/77.1	65	20
18	TPeP	19.40	309.0/99.0, 309.0/169.0	67	30
19	BDP	19.95	693.0/367.1, 693.0/327.1	180	45
20	TXP	20.07	411.0/179.0, 411.0/194.0	145	51
21	IPPP	20.20	453.2/327.2, 453.2/369.3	145	45
22	TBPP	20.20	438.8/327.2, 438.8/383.2	145	40
23	THP	20.30	351.2/99.0, 351.2/267.2	76	30
24	TiPPP	20.44	453.1/327.1, 453.1/369.1	126	42
25	TOP	21.48	435.3/99.1, 435.3/211.0	76	42

**Table 3:** Linear equations, linear ranges, correlation coefficients (R^2^) and method detection limits (MDL) of OPEs.

Compounds	Retention Times/min	ME	Linear Ranges ng/mL	R^2^	Linear Equations	MDL (ng/g)	LQD (ng/g)
TMP	1.00	0.99	1–100	0.99880	Y = 0.11265X + 0.0700	0.02	0.08
TEP	1.26	1.04	1–100	0.99612	Y = 0.18548X + 0.02390	0.02	0.08
TCEP	1.36	1.04	5–100	0.99875	Y = 0.00769X + 0.01530	0.12	0.48
TPPO	2.04	0.92	1–100	0.99554	Y = 0.21752X + 0.024626	0.02	0.08
TCPP	2.50	0.96	5–100	0.99853	Y = 0.01990X + 0.00439	0.60	2.40
TPrP	2.55	1.07	1–100	0.99595	Y = 0.09954X + 0.02078	0.01	0.04
TDCP	4.67	1.17	5–100	0.99717	Y = 0.00246X − 0.00363	0.80	3.20
TPP	4.90	1.15	5–100	0.99883	Y = 0.57701X + 1.62750	0.14	0.64
TZ3P	6.53	1.09	10–100	0.99475	Y = 0.00167X − 0.00484	1.08	4.32
TnBP	7.68	1.13	1–100	0.99886	Y = 4.04018X + 1.07832	0.01	0.04
CDP	7.30	1.09	5–100	0.99689	Y = 0.05552X + 0.01985	0.55	2.20
TiBP	7.14	1.06	1–100	0.99693	Y = 3.06331X + 0.96103	0.02	0.08
TBEP	10.36	1.18	1–100	0.99565	Y = 0.89769X + 0.38928	0.03	0.12
p-TCP	7.90	1.13	5–100	0.99835	Y = 0.5897X + 0.56539	0.08	0.32
o-TCP	15.09	1.14	5–100	0.99694	Y = 0.59529X + 0.54642	0.08	0.32
m-TCP	15.46	1.08	10–100	0.99861	Y = 0.56601X + 0.55150	0.09	0.36
EHDPP	19.24	1.19	20–100	0.99544	Y = 0.20223X + 0.41684	0.13	0.52
TPeP	19.40	1.05	5–100	0.99543	Y = 1.11483X + 3.86615	0.05	0.20
BDP	19.95	1.14	5–100	0.99963	Y = 0.08851X − 0.02858	0.19	0.76
TXP	20.07	1.12	5–100	0.99571	Y = 0.28008X + 0.83879	0.07	0.28
IPPP	20.20	1.11	1–100	0.99751	Y = 2.33449X + 1.04205	0.05	0.20
TBPP	20.20	1.09	5–100	0.99955	T = 0.7255X + 1.85226	0.07	0.28
THP	20.30	1.08	1–100	0.99680	Y = 2.31602X + 63.65371	0.05	0.20
TiPPP	20.44	1.13	1–100	0.99951	Y = 1.83202 + 16.87611	0.05	0.20
TOP	21.48	1.03	1–100	0.99899	Y = 0.53942X + 1.46183	0.03	0.12

**Table 4:** Results of tests for precision and spiked recoveries (*n* = 6).

Compound	Low		Medium		High	
	Recovery%	RSD%	Recovery%	RSD%	Recovery%	RSD%
TMP	71.3	10.6	76.2	6.9	79.5	6.6
TEP	72.9	11.8	83.1	8.5	78.0	7.9
TCEP	74.3	3.6	72.5	3.3	78.4	3.1
TPPO	80.7	2.3	80.1	2.0	78.8	1.2
TCPP	83.5	6.6	89.5	5.4	84.8	2.2
TPrP	107	3.5	109	2.0	111	1.9
TDCP	99.2	3.9	97.9	6.1	97.5	3.1
TPP	90.1	2.0	91.2	1.6	90.6	1.8
TZ3P	102	8.5	108	6.3	105	4.6
TnBP	98.6	6.0	103	3.2	95.4	2.0
CDP	88.4	5.9	96.5	5.3	95.1	4.5
TiBP	103	3.6	98.3	1.9	100	1.8
TBEP	92.5	4.6	98.7	1.7	96.0	2.3
p-TCP	91.7	5.2	92.6	1.6	92.0	1.5
o-TCP	105	2.9	105	2.7	110	2.7
m-TCP	88.8	5.4	98.6	5.1	102	3.5
EHDPP	91.2	3.3	94.9	3.8	92.2	3.0
TPeP	98.9	4.4	98.7	2.1	99.8	2.0
BDP	83.4	6.6	85.0	5.5	80.9	4.9
TXP	75.2	3.1	79.7	2.9	83.6	2.1
IPPP	70.6	4.8	76.3	3.1	77.4	2.7
TBPP	79.6	4.9	80.3	5.2	80.2	4.0
THP	103	4.4	99.1	4.0	98.3	3.0
TiPPP	90.1	4.7	89.9	4.4	91.4	2.6
TOP	88.1	7.9	93.3	6.6	95.8	5.3

**Table 5:** Analytical results of samples.

Compounds	Sample 1 (ng/g)	Sample 2 (ng/g)	Sample 3 (ng/g)	Sample 4 (ng/g)
TMP	ND	ND	ND	ND
TEP	0.517	0.502	0.532	0.534
TCEP	0.476	0.603	0.233	0.489
TPPO	0.414	0.374	0.535	0.537
TCPP	10.4	11.4	8.84	8.34
TPrP	ND	ND	ND	ND
TDCP	ND	ND	0.751	0.348
TPP	ND	ND	ND	ND
TZ3P	ND	ND	ND	ND
TnBP	ND	ND	ND	ND
CDP	ND	ND	ND	0.304
TiBP	ND	ND	ND	ND
TBEP	0.0445	0.00800	ND	ND
p-TCP	ND	ND	ND	ND
o-TCP	ND	ND	ND	ND
m-TCP	ND	ND	ND	ND
EHDPP	ND	ND	ND	ND
TPeP	ND	ND	ND	ND
BDP	0.678	0.678	1.75	1.56
TXP	0.261	0.146	0.21	0.0350
IPPP	ND	ND	ND	ND
TBPP	ND	ND	ND	ND
THP	0.448	0.421	0.215	0.162
TiPPP	0.041	0.0510	0.0085	ND
TOP	1.980	2.51	ND	ND

ND, not detected.

## Data Availability

The raw data supporting the conclusions of this article will be made available by the authors on request.

## References

[1] Han X., Li W., Liu J., Wu C., Zhang Y., Pei S. (2022). Controlling techniques and characteristics of organophosphate esters in building environment: A review. Chin. J. Eng..

[2] Fernández-Arribas J., Moreno T., Eljarrat E. (2023). Human exposure to organophosphate esters in water and packed beverages. Environ. Int..

[3] Pei J., Dong X., Zhang J. (2024). Levels, profiles and human exposure of organophosphate esters (OPEs) in dust from subway sta-tions. Build. Environ..

[4] Zeng X., He L., Cao S., Ma S., Yu Z., Gui H., Sheng G., Fu J. (2014). Occurrence and distribution of organophosphate flame retardants /plasticizers in wastewater treatment plant sludges from the Pearl River Delta China. Environ. Toxicol. Chem..

[5] Li J., Yu N., Zhang B., Jin L., Li M., Hu M., Zhang X., Wei S., Yu H. (2014). Occurrence of organophosphate flame retardants in drinking water from China. Water Res..

[6] Wang R., Tang J., Xie Z., Mi W., Chen Y., Wolschke H., Tian C., Pan X., Luo Y., Ebinghaus R. (2015). Occurrence and spatial distribution of organophosphate ester flame retardants and plasticizers in 40 rivers draining into the Bohai Sea, Environmental North China. Pollution.

[7] Shi Y., Gao L., Li W., Wang Y., Liu J., Cai Y. (2016). Occurrence, distribution and seasonal variation of organophosphate flame retardants and plasticizers in urban surface water in Beijing, China. Environ. Pollut..

[8] He M.J., Yang T., Yang Z.H., Li Q., Wei S.-Q. (2017). Occurrence and distribution of organophosphate esters in surface soil and street dust from Chongqing, China: Implications for human exposure. Arch. Environ. Contam. Toxicol..

[9] Luo Q., Shan Y., Muhammad A., Wang S., Sun L., Wang H. (2018). Levels, distribution, and sources of organophosphate flame retardants and plasticizers in urban soils of Shenyang, China. Environ. Sci. Pollut. Res..

[10] Cao S., Zeng X., Song H., Li H., Yu Z., Sheng G., Fu J. (2012). Levels and distributions of organophosphate flame retardants andplasticizers in sediment from Taihu Lake, China. Environ. Toxicol. Chem..

[11] Zheng J., Gao Z., Yuan W. (2014). Development of pressurized liquid extraction and solid-phase microextraction combined with gas chromatography and ame photometric detection for the determination of organophosphate esters in sediments. J. Sep. Sci..

[12] Tajima S., Araki A., Kawai T., Tsuboi T., Bamai Y.A., Yoshioka E., Kanazawa A., Cong S., Kishi R. (2014). Detection and intake assessment of organophosphate flame retardants in house dust in Japanese dwellings. Sci. Total Environ..

[13] Salamova A., Hermanson M., Hites R.A. (2014). Organophosphate and halogenated flame in atmospheric particles from a European Arctic site. Environ. Sci. Technol..

[14] Feng L., Ouyang F., Liu L., Wang X., Wang X., Li Y.-J., Murtha A., Shen H., Zhang J., Zhang J.J. (2016). Levels of urinary metabolites of organophosphate flame retardants, TDCIPP, and TPHP, in pregnant women in Shanghai. J. Environ. Public Health.

[15] Sun Y., Gong X., Lin W., Wang Y., Wu M., Kannan K., Ma J. (2018). Metabolites of organophosphate ester flame retardants in urine from Shanghai, China. Environ. Res..

[16] Zhao F., Wan Y., Zhao H., Hu W., Mu D., Webster T.F., Hu J. (2016). Levels of blood organophosphorus flame retardants and association with changes in human sphingolipid homeostasis. Environ. Sci. Technol..

[17] Ma Y., Jin J., Li P., Xu M., Sun Y., Wang Y., Yuan H. (2017). Organophosphate ester flame retardant concentrations and distributions in serum from inhabitants of Shandong, China, and changes between 2011 and 2015. Environ. Toxicol. Chem..

[18] He C., Toms L.L., Thai P., Eede N.V.D., Wang X., Li Y., Baduel C., Harden F.A., Heffernan A.L., Hobson P. (2018). Urinary metabolites of organophosphate esters:Concentrations and age trends in Australian children. Environ. Int..

[19] Yusa V., Ye X., Calafat A.M. (2012). Methods for the determination of biomarkers of exposure to emerging pollutants in human specimens. Trends Anal. Chem..

[20] Ding J., Xu Z., Huang W., Feng L., Yang F. (2016). Organophosphate ester flame retardants and plasticizers in human placenta in eastern China. Sci. Total Environ..

[21] Amanda M.R., Amy H.H., Gro D.V., Cathrine T., Amrit K.S., Enrique C., Heidi A., Stephanie M.E. (2023). The association of prenatal phthalates, organophosphorous pesticides, and organophosphate esters with early child language ability in Norway. Environ. Res..

[22] Yang P., Xie J., Huang S., Li X., Deng L., Zhang J., Chen L., Wu N., Huang G., Zhou C. (2023). Cocktail” of environmental chemicals and early reproductive out-comes of IVF: The insight from paternal and maternal exposure. Environ. Manag..

[23] Liu S.L., Zhang H., Hu X.H., Qiu Y.-L., Zhu Z.-L., Zhao J.-F. (2016). Analysis of organophosphate esters in sediment samples using gas chromatography-tandem mass spectrometry. Chin. J. Anal. Chem..

[24] Liu J., Zeng X.Y., Yu Z.Q., Sheng G.Y., Fu J.M. (2016). Determination of synthetic musks and organophosphate flame retardants/plasticizers in solid phase by ultrasound assisted extraction coupled with solid phase extraction. J. Instrum. Anal..

[25] Luo Q., Wang S., Sun L., Wang H. (2018). Simultaneous accelerated solvent extraction and purification for the determination of 13 organo-phosphate esters in soils by gas chromatography-tandem mass spectrometry. Environ. Sci. Pollut. Res..

[26] García-López M., Rodríguez I., Cela R. (2009). Pressurized liquid extraction of organophosphate triesters from sediment samples using aqueous solutions. J. Chromatogr. A.

[27] Wang C.Y., Li X., Yang Z.J., Lin J.F., Ji H.L., Li W.H. (2020). Simultaneous determination of thirteen organophosphate retardants in lith-ium-ion battery electrolyte by gas chromatography-tandem mass spectrometry. J. Instrum. Anal..

[28] Ni H.P., Liu X.P., Li D.K., Zhang Y. (2022). Determination of 11 organophosphorus flame retardants in edible vegetable oil by gel permeation chromatography gas chromatography-tandem mass spectrometry. J. Chin. Cereal Oils Assoc..

[29] Li Y.Q., Li W., Zhang R.R., Zhang Z.M., Sun A.L., Shi X.Z. (2020). Determination of organophosphorus flame retardants in marine fish by QuEChERS-gas chromatography tandem mass spectrometry. Chin. J. Anal. Lab.

[30] Pan M., Tong L., Tian Q., Song S. (2023). Determination of 15 Organophosphate Ester Flame Retardants in Soils and Sediments by Gas Chromatography-Mass Spectrometry with Accelerated Solvent Extraction. Rock. Mineral. Anal..

[31] Wang T., Zhang H., Zhou H.L., Lin J., Tong P., Ben Y., Wang Y., Han Y., Zhang Y., Li J. (2023). Simultaneous determination of 12 organophosphate esters in soil by solid phase ex-traction/high performance liquid chromatography-tandem mass spectrometry. J. Instrum. Anal..

[32] Dankyi E., Gordon C., Carboo D., Fomsgaard I.S. (2014). Quantification of neonicotinoid insecticide residues in soils from cocoa plantations using a QuEChERS extraction procedure and LC-MS/MS. Sci. Total Environ..

[33] Cristale J., Lacorte S. (2013). Development and validation of a multiresidue method for the analysis of polybrominated diphenyl ethers, new brominated and organophosphorus flame retardants in sediment, sludge and dust. J. Chromatogr. A.

[34] Guo X., Mu T., Xian Y., Luo D., Wang C. (2016). Ultra-performance liquid chromatography tandem mass spectrometry for the rapid simultaneous analysis of nine organophosphate esters in milk powder. Food Chem..

[35] Yang J., Zhou X., Li X., Li X., Gao Y., Zhang Q. (2023). Simultaneous determination of 21 organophosphorus flame retardants in rice by gas chromatography quadrupole time-of-flight mass spectrometry. Talanta.

[36] Cui K., Wen J., Zeng F., Zhou X., Li S., Zeng Z. (2017). Determination of organophosphate ester flame retardants and plasticizers in soil samples by microwave-assisted extraction coupled with silica gel/alumina multilayer solid phase extraction and gas chromatography-mass spectrometry. Anal. Methods.

[37] Tian Q., Tong L., Pan M., An Z., Xu C., Zhang M. (2025). Simultaneous determination of phthalate esters and organophosphate esters in soil by isotopic dilution-gas chromatography-mass spectrometry. Phys. Test. Chem. Anal. Part B Chem. Anal..

[38] Lu J.-X., Ji W., Ma S.-T., Yu Z.-Q., Wang Z., Li H., Ren G.-F., Fu J.-M. (2014). Analysis of Organophosphate Esters in Dust, Soil and Sediment Samples Using Gas Chromatography Coupled with Mass Spectrometry. Chin. J. Anal. Chem..

[39] Leon N., Pastor A., Yusa V. (2016). Target analysis and retrospective screening of veterinary drugs, ergot alkaloids, plant toxins and other undesirable substances in feed using liquid chromatography-high resolution mass spectrometry. Talanta.

[40] Cui K.Y., Wen J.X., Zeng F., Li S., Zhou X., Zeng Z. (2017). Occurrence and distribution of organophosphate esters in urban soils of the subtropical city, Guangzhou, China. Chemosphere.

[41] Wang T., Zhang H., Huang C., Ben Y., Zhou H., Guo H., Han Y., Zhang Y., Tong P. (2025). Occurrence and potential risks of organophosphate esters in agricultural soils: A case study of Fuzhou City, Southeast China. J. Environ. Sci..

[42] van der Veen I., de Boer J. (2012). Phosphorus flame retardants: Properties, production, environmental occurrence, toxicity and analysis. Chemosphere.

[43] Xu H., Wang Z., Zhang S., Guo M., Chen M., Shi L. (2018). Research progress on toxicity effects of organophosphate flame retardants. Asian J. Ecotoxicol..

[44] Peng B., Yu Z.M., Wu C.C., Liu L.-Y., Zeng L., Zeng E.Y. (2020). Organophosphate esters in human hair from South China: Levels, distribution, and human exposure. Environ. Int..

[45] Li X.H., Ma J., Fang D., Shi T., Gong Y. (2020). Organophosphate flame retardants in soils of Zhejiang Province, China: Levels, distribution, and possible sources. Arch. Environ. Contam. Toxicol..

[46] Yao C., Yang H., Li Y. (2021). A review on organophosphate flame retardants in the environment: Occurrence, accumulation, metabolism and toxicity. Sci. Total Environ..

[47] Yang Y., Liu P., Li M. (2020). Tri-n-butyl phosphate induced earthworm intestinal damage by influencing nutrient absorption and energy homeostasis of intestinal epithelial cells. J. Hazard Mater..

[48] Sang Y., Li W., Liu J., Chu C., Zhao J., Cai H., Hong S. (2020). Simultaneous determination of 13 organophosphate esters in greenhouse soil by gas chromatography-mass spectrometry. J. Instrum. Anal..

